# Tissue Competence Imprinting and Tissue Residency of CD8 T Cells

**DOI:** 10.3389/fimmu.2013.00283

**Published:** 2013-09-17

**Authors:** Roman Krzysiek, Marie-Ghislaine de Goër de Herve, Heng Yang, Yassine Taoufik

**Affiliations:** ^1^Department of Immunology, CHU Bicêtre, Le Kremlin-Bicêtre, France; ^2^INSERM U-996, Clamart, France; ^3^INSERM U-1012, Le Kremlin-Bicêtre, France

**Keywords:** effector T cells, memory T cells, tissue-specific imprinting, tissue residency

## Abstract

T cell immunity is characterized by striking tissue specialization. Tissue-specificity imprinting starts during priming by tissue-derived migratory dendritic cells in the non-random, specialized micro-anatomical area of the draining lymph node and is influenced by constitutive and induced cues from local environment. Besides tissue-specific effectors, memory cells also exhibit a tissue-specificity. Long-lived tissue-resident memory T cells likely play a considerable role in preventing pathogen invasion. Understanding of the mechanisms of tissue specialization of T cells is of major importance for the design of optimal vaccination strategies and therapeutic interventions in tissue/organ-specific inflammatory diseases. The present review summarizes our current knowledge and hypothesis about tissue-specificity imprinting and tissue residency of T cells.

Generation of long-lasting systemic and tissue-specific memory T cell responses is a fundamental characteristic of adaptive immunity. According to the current paradigm, naturally pre-programed and secondary lymphoid tissue (SLT)-oriented trafficking of recirculating naïve T cells switches dramatically to peripheral tissue-oriented after antigen (Ag) exposure and priming in the T cell zone environment of draining lymph node (DLN) (1). The role of peripheral tissue-derived migratory dendritic cells (DCs) in the imprinting of tissue-specific information is now well documented (2–5). Thus, DLNs can be considered as the body’s “central processing units” incorporating tissue-specific information into generated adaptive T cell responses. This response is generated from two recirculating precursors characterized by constitutive LN-tropism: naive and central memory (T_CM_) T cells. Indeed, both populations share high expression of the LN-homing molecules (CCR7 and CD62L) and recirculation marker S1P1 and generate tissue-specific short-lived effector cells (SLECs), the major pool of terminally differentiated T cells highly active in pathogen clearance. Although the role of CD8α+ resident DCs in generation of tissue-specific SLECs in some types of infections has been documented (6), peripheral tissue-derived migratory DCs (CD207^+^CD103^+^ and CD11b^+^CD207^−^CD103^−^dermal DCs and CD207^+^CD103^−^ Langerhans cells, LCs) likely represent major players involved in processing and transfer of skin-specific information during priming and cross-priming processes (6, 7).

Recent multiparameter 3D imaging approach revealed striking differences in migratory DCs-subset distribution within discrete micro-anatomical compartments of skin DLN with preferential localization of CD11b^+^ dermal DCs within interfollicular and outer paracortex area close to B cell zone and preferential positioning of dermal CD207^+^CD103^+^ DCs and LCs within deeper T cell zone (8). This non-random spatial segregation and positioning of tissue-derived DCs subpopulations in DLN mirrors controlled trafficking and positioning of naive and T_CM_ cell subsets following priming and during recall T cell response, respectively. In contrast to steady state where naive CD8^+^ T cells localize mostly in the deep T cell area, recent observations point to the crucial role of subfollicular region previously known as cortical ridge and interfollicular area (IFA) as preferential sites of both, positioning following CD8^+^ T cell priming and constitutive prepositioning of CD8^+^ T_CM_ cells (9, 10). Notably, in both cases, tissue-specific SLECs are generated, which suggest a striking spatial overlap between local DLN functional microdomain involved in generation of SLECs progeny regardless of the type of adaptive immune response (primary or secondary). CXCL9-CXCR3 receptor chemotactic axis has been identified to be a key mechanism regulating repositioning of T_CM_ to cortical ridge zone and IFA and CXCR3 has been proposed to be a factor involved in early programing of CD8^+^ T cells differentiation into SLECs since CXCR3^−/−^ CD8^+^ T cells preferentially differentiate into memory precursor cells (MPECs) (9, 11, 12). Importantly, CXCR3 is a functional marker shared by recently activated naïve T cells, SLECs, and different subsets of memory T cells including the recently identified memory stem cell subset (T_SCM_) (13). Whether re-stimulation of T_SCM_ cells also takes place in the cortical ridge zone and IFA remains to be established.

Tissue-specific imprinting during T cell priming and subsequent T cell differentiation into tissue-specific SLECs is thought to involve induction of a set of surface homing molecules which combinatorial usage generates lymphocyte’s tissue-specific zip code. Only a few molecular mechanisms involved in the peripheral tissue tropism of SLECs have so far been identified. Small-intestine tropism of SLECs is imprinted by gut-associated DCs in an all-trans retinoic acid (RA)-dependent manner. This involves induction of a small intestine-specific zip code consisting of strong integrin α4β7 and chemokine receptor CCR9 expression (4, 14). Likewise, skin-derived DCs induce chemokine receptor CCR10 expression on T cells in a 1,25(OH)_2_D_3_-dependent manner and thereby program generated SLECs to home to the skin (15). The skin-specific zip code is characterized by concomitant and sequential usage of E-/P-selectin ligands (CLA), CCR4, CCR10, CCR8, CCR6, αMβ2 (CD11b/CD18), and α4β1 (CD49d/CD29), among other homing molecules (16). *De novo* expression of E-selectin ligands on skin-tropic T cells requires activation-induced expression of the α(1,3)fucosyltransferases FucT-IV and FucT-VII glycosylation enzymes (17, 18). Of note, expression of skin-tropic molecules on lymphocytes is inhibited by RA, while 1,25(OH)_2_D_3_ negatively regulates RA-controlled expression of α4β7 and CCR9 (14). The molecular mechanisms that control homing of effector T cells to other tissues remain largely unknown except of the recently proposed role of CLEVER-1/stabilin-1 and VAP-1 in liver-specific recruitment of Foxp3^+^ Tregs (19).

The lymphocyte tissue-specific zip codes in turn recognize yet poorly defined vascular zip codes, a combinatorial set of endothelial counter receptors for lymphocyte’s zip codes expressed by post-capillary venules. Importantly, both types of zip code may modify through induction of new surface markers and down regulation of others, thus adapting cell trafficking to the type of immune response and to dynamically changing inflammatory conditions. The last notion is indeed supported by substantial heterogeneity and dynamics of microvascular endothelial phenotype described within the same tissue compartment in response to inflammatory stimuli (20). Phenotypic and functional modifications of local post-capillary endothelial cells (ECs) induced by pro-inflammatory cytokines and inflammation-related factors, likely includes: (i) rearranged expression of adhesion molecules, glycosaminoglycan (GAGs) structures, and specific glycosylation patterns of the endothelial cell surface-associated molecules, (ii) active transport, transcytosis, and presentation of inflammatory chemokines induced at sites of inflammation, at the abluminal surface of endothelial cells, (iii) synthesis of lymphocyte-activating cytokines including chemokines (21–23). Thus, although organ/tissue-specificity seems to be pre-determined in the context of DLNs, precise tissue-specific positioning of SLECs seems to be characterized by sequential modifications of the expressed zip codes which are dynamically adjusted according to the given step of cell trafficking process and local inflammatory and metabolic cues.

Despite clear evidence for the imprinting of a specific homing pattern during T cell priming, this elegant model likely has certain limitations, as several experimental studies of infectious diseases have shown wider recirculation pattern and substantial promiscuity in the trafficking programing, as well as a surprisingly flexible tissue distribution of *in vivo*-induced SLECs (24–26). However, large or biased homing patterns observed in some models could be due to several immune and pathogen-related factors: (i) *in vivo* model used, (ii) artifacts related to high number transfer of Ag-specific transgenic T cells (iii) low level of induction of broad-spectrum homing molecules such as mucosal T cells-associated marker α4β7 integrin or inflammation-related molecules (iv) immunization route (v) priming such as Ag load, priming threshold, adjuvants and costimulation, polarization, or regulatory signals, and (vi) target tissue-analyzed (more promiscuous, mucosa-associated, and more restricted skin-specific homing pattern), among others (24–29).

While the mechanisms controlling homing pattern of SLECs has been extensively investigated, less attention has been paid to the mechanisms that control the trafficking and tissue positioning of long-lived memory T cell subsets, namely peripheral tissue-resident memory (T_RM_) T cells. The simultaneous presence of memory T cells with different anatomical distributions (systemic and tissue/organ-specific) is likely to reflect the role of compartmentalization in the global strategy used by the immune system to ensure optimal surveillance and protection (Figure 1). In this scenario, similarly to SLECs, T_EM_ cells do not express the LN-homing molecules CD62L and CCR7, and thus traffic preferentially to inflammatory sites of Ag encounter. However, a subset of T_EM_ cells likely can egress from peripheral inflammatory sites to local DLN via afferent lymph, following transient expression of CCR7, and then to the bloodstream and spleen (30–32). The proportion of cells expressing CD62L within the circulating memory T cell population increases with increasing time after immunization. This could be related to the higher survival and homeostatic proliferation of T_CM_ relative to T_EM_ (29). T_CM_ cells continually re-circulate between peripheral blood and SLT and account for the bulk of long-lasting systemic immune memory and are the most important precursors of tissue-specific T_EM_ pool during recall response. However, they are only moderately capable of homing to sites of inflammation, and their ability to control localized infections within peripheral tissues is thus very limited (33–36).

**Figure 1:**
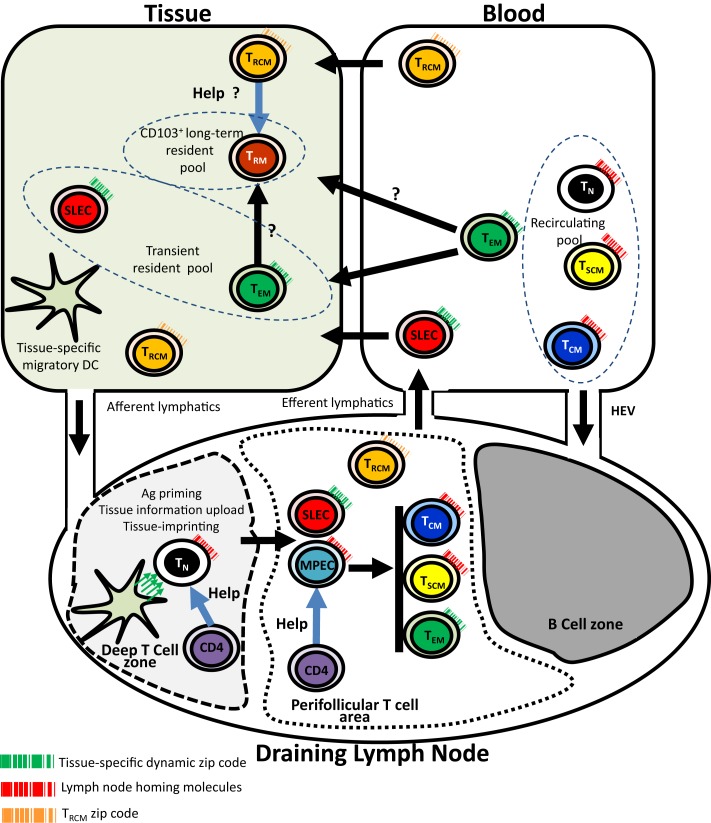
**Tissue-specific imprinting, trafficking, and tissue residency of effector and memory CD8 T cells**. In this model, tissue-derived migratory DC imprints tissue-specific homing pattern (symbolized by the green arrows) in naive T cell during priming in the context of the deep T cell zone of the draining lymph node. Primed T cells including SLECs and MPECs relocalize into perifollicular T cell area. While SLECs migrate into tissues through the blood, MPECs complete their differentiation into memory subsets. Recirculating (T_N_, T_CM_, T_SCM_), transient (SLECs, T_EM_), and long-term (T_RM_) non-recirculating CD8 T cell pools are shown. Unusual trafficking pattern of CD4^+^ T_RCM_ is also shown. CD4 helping signals for CD8 memory generation and possibly for T_RM_ maintenance are also shown. T_N_, naive CD8 T cell; SLEC, short-lived effector T Cell; MPEC, memory precursor CD8 T cell; T_SCM_, stem cell memory CD8 T cell; T_CM_, central memory CD8 T cell; T_EM_, effector memory CD8 T cell; T_RM_, resident memory CD8 T cell; T_RCM_, recirculating memory CD4 T cell; HEV, high endothelial venules.

Tissue-resident memory cells in contrast are non-recirculating long-lived pathogen-specific resident memory T cells permanently associated with body surfaces (skin, intestine, lung, vaginal mucosa) and with organs such as the brain, kidney, and pancreas, thus providing global organ/area-targeted protection (34, 37, 38). Skin T_RM_ cells have potent effector capacities and appear be superior to recirculating T_CM_ cells at providing rapid long-term protection against cutaneous re-infection (34). Several data indicate that local environmental cues are required for T_RM_ cell maintenance within epithelia, as described for LC (38–41). According to this scenario, locally produced TGFβ could instruct specific precursors within the T_EM_ pool to follow a T_RM_ fate. Some T_RM_ populations have been described to constitutively express CD8αα homodimer (42). A possible hypothesis is that T_RM_ precursors require CD8αα and the interaction with its high affinity ligand(s) such as thymic leukemia antigen (TL) for their differentiation and survival, as described for intestinal intraepithelial lymphocytes (IELs) (43).

Currently, there are no data supporting the hypothesis that the T_RM_ pool results from seeding by blood-borne T_CM_-derived progeny. In contrast, it has been shown that T_EM_ generated following skin infection accumulate as T_RM_ in a organ-specific manner both, at site of infection and at distant sites (34). This suggests that the T_EM_ stage is the link between migratory DC_S_ that harbor tissue-specific information and T_RM_ cells. This implies that the tissue-specific information is conserved and transmitted along this T_EM_–T_RM_ differentiation pathway. However, T_RM_ cells differ markedly from blood T_EM_ pool and the exact relations between T_EM_ and T_RM_ is yet to be determined, especially whether T_RM_ may be viewed only as a resident form of blood-derived T_EM_ (38, 44–46). Commonly observed signature of the barrier tissue-associated T_RM_ cell population include strong expression of CD103, E-/P-selectin ligands, CD44, CD69 (a negative regulator of recirculation marker S1P1) (47), and CD49a/VLA-1. This phenotype is partially shared with other E-cadherin-dependent epithelia-resident cells such as dendritic epidermal γδ T cells (DETC) and IELs (35, 48). High level of CD103 expression likely represents a key marker of permanent barrier tissue residency of different types of T_RM_ subsets (42, 49). The phenotype of T_RM_ cells is probably much more heterogeneous, and future studies will likely identify new molecules involved in T_RM_ cell positioning and maintenance within barrier as well as non-barrier peripheral tissues. Several observations suggest that T_RM_ cells can persist long-term without any external input (33, 44, 45, 49), therefore providing prolonged protection against pathogens. However, the observations of numerous cases of progressive multifocal leukoencephalopathy (a devastating demyelinating disease due to JC virus reactivation in the brain) in multiple sclerosis patients treated with natalizumab (50), a therapeutic monoclonal antibody directed against the cell adhesion molecule α4-integrin that blocks T cell trafficking through the blood brain barrier, raises question about the long-term maintenance of efficient brain-resident T cell memory without cell input from peripheral blood.

A point of interest is the recent demonstration in the skin of recirculating memory (T_RCM_) CD4 T cells which phenotypically differ from T_EM_, as they are CCR7^pos^, CD62L^int^, CD103^±^ in addition to the expression of the peripheral homing receptor E-selectin ligand (51). This unusual migration pattern is partially shared by Foxp3^+^ Tregs that migrate from skin to draining LNs via afferent lymphatics (52). Following activation, T_RCM_ expressed CD40L and produced IL-2 (51). Numerous reports have shown the requirement of helping signals from CD4 T cell subsets for the generation, maintenance, and reactivation of efficient systemic CD8 memory (53–57). Whether help from CD4 T_RCM_ is required for the maintenance of CD8 T_RM_ is a point that deserves further investigations. Such a requirement of help from recirculating CD4 T cells may provide an explanation for the deleterious role of natalizumab or efalizumab (another therapeutic monoclonal antibody that targets the member of the integrin family CD11a) (50) on brain-resident CD8 memory, despite the intrinsic long-term survival of CD8 T_RM_ cells. Another question of interest is whether there is a CD8 counterpart of CD4 T_RCM_ cells. Figure 1 summarizes key points of our current understanding of trafficking and tissue residency of effector and memory T cell subsets.

We are still at the early beginning of the story and many questions remain unanswered about the mechanisms of tissue-specific imprinting of effector and memory T cells and the maintenance of resident T cell memory as well as the lineage relationships between T_RM_ and blood memory T cells. Unraveling those mechanisms is of major importance for the design of new vaccination strategies capable of inducing strong cell-mediated organ/tissue-specific immunity and protection. Molecules involved in tissue/organ-specific positioning of memory T cells might also represent interesting therapeutic targets for a variety of tissue/organ-specific inflammatory diseases.

## Conflict of Interest Statement

The authors declare that the research was conducted in the absence of any commercial or financial relationships that could be construed as a potential conflict of interest.
